# Imaging biomineralizing bacteria in their native-state with X-ray fluorescence microscopy†

**DOI:** 10.1039/d4sc08375j

**Published:** 2025-03-03

**Authors:** Daniel M. Chevrier, Elisa Cerdá-Doñate, Lucía Gandarias, Miguel A. Gomez-Gonzalez, Sufal Swaraj, Paul E. D. Soto-Rodriguez, Antoine Fraisse, Tom Robinson, Damien Faivre

**Affiliations:** a Université Aix-Marseille, CNRS, CEA, BIAM, UMR7265 Institut de Biosciences and Biotechnologies d’Aix-Marseille CEA Cadarache Saint-Paul-lez-Durance F-13108 France daniel.chevrier@cea.fr; b Max Planck Institute of Colloids and Interfaces, Department Biomaterials Potsdam 14424 Germany; c Max Planck Institute of Colloids and Interfaces, Department Theory & Biosystems Potsdam 14424 Germany; d Depto. Electricidad y Electrónica, Universidad del País Vasco (UPV/EHU) Leioa 48940 Spain; e Diamond Light Source, Harwell Science and Innovation Campus Didcot OX11 0DE UK; f SOLEIL Synchrotron, L'Orme des Merisiers Départementale 128 Saint-Aubin 91190 France; g Instituto de Estudios Avanzados IUDEA, Departamento de Física, Universidad de La Laguna C/Astrofísico Francisco Sánchez, s/n., Tenerife E-38203 Spain; h Institute for Bioengineering, School of Engineering, University of Edinburgh Edinburgh EH9 3DW UK; i Department of Physics, University of Latvia Jelgavas iela 3 LV-1004 Latvia

## Abstract

Understanding the interactions between metal-based nanoparticles and biological systems in complex environments (*e.g.*, the human body, soils, and marine settings) remains challenging, especially at single-cell and nanoscale levels. Capturing the dynamics of these interactions, such as metal distribution, nanoparticle growth, or degradation, in their native state (*in vivo*) is particularly difficult. Here, we demonstrate the direct measurement of iron content in hydrated, magnetite-biomineralizing magnetotactic bacteria using synchrotron-based nanobeam-scanning X-ray fluorescence microscopy combined with a liquid cell environment. In addition to X-ray fluorescence imaging, we collected iron chemical speciation information from individual bacteria in liquid using X-ray absorption spectroscopy. To follow biomineralization *in situ*, we developed a microfluidic device to track magnetite nanoparticle formation over several hours under the X-ray beam. This approach highlights the potential of X-ray fluorescence microscopy in liquid cell setups to provide elemental and chemical insights into biological processes at the single-cell level. Combining X-ray nanobeam techniques with liquid cell devices will enable more “on-chip” experiments on metals in biological contexts to be conducted at the synchrotron.

## Abstract 2

Using synchrotron nanobeam X-ray fluorescence microscopy with a liquid cell, intracellular iron is quantified in hydrated magnetotactic bacteria, and magnetite biomineralization is tracked *in situ*, advancing metal imaging in biological systems.

## Graphical abstract

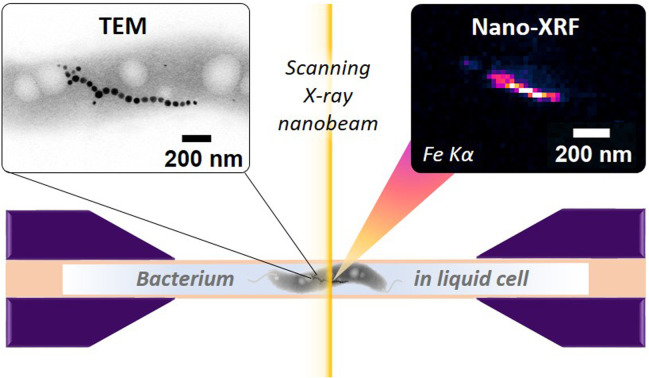

## Introduction

Having access to chemical and structural information at the nanoscale is critical to investigate a number of persistent scientific questions in life science research, such as how metallo-drugs enter and fight diseased cells, how cells interact with nanomaterials or nanoplastics, and how biomineralized materials are constructed.^1–3^ Analytical techniques that reveal this information at the nanoscale are commonly electron-based and X-ray-based microscopies. X-ray microscopy has an advantage over electron microscopy for studying hydrated biological samples in that X-rays have weaker interactions with matter than electrons, thus having higher penetration depth of the sample.^4,5^ This can also lead to less damage transferred to the biological system.^6^ X-ray microscopy, in particular microbeam- or nanobeam-scanning techniques, is multimodal (combined scattering, fluorescence and transmission detection) with a tunable incident X-ray energy that allows for chemistry- and element-specific imaging techniques.^7^

Synchrotron-based X-ray microscopy techniques are making important advancements in life science research as they can not only extract elemental and structural information of individual cells with subcellular resolution (<100 nm), but also in three dimensions and under near native-state conditions (*i.e.*, ice-vitrified).^1,8–11^ However, lengthy sample preparation and low-throughput are still bottlenecks for conducting extensive near native-state studies of frozen, hydrated biological specimens.^9,12–14^ An ambitious alternative is to perform measurements on whole, hydrated cells under ambient conditions, capturing their intracellular composition or biological state *in vivo*. Although the field of liquid cell transmission electron microscopy (TEM) is well developed, its use is limited to the study of thin materials (*i.e.*, only a few hundred nm in thickness).^15^ A few studies have imaged individual biological cells in liquid using X-ray microscopy,^16–19^ but do not follow biological processes *in situ*. As a result, there are few suitable liquid cell environments known to be interfaced with X-ray microscopy techniques to enable native-state imaging and *in situ* experimentation of biological systems.

In this work, nanobeam-scanning X-ray fluorescence microscopy (nano-XRF) is employed to measure the intracellular iron content of living magnetotactic bacteria (MTB) in a liquid cell environment. Magnetotactic bacteria were used in this work as they produce chains of magnetite (Fe_3_O_4_)-containing organelles called magnetosomes that can be detected by X-ray fluorescence (XRF) using a scanning X-ray nanobeam as shown in previous work.^20,21^ Nanobeam-scanning X-ray fluorescence microscopy is used as a semi-quantitative technique to determine metal concentrations of flat, dry samples.^22^ In this study, due to the complexity of a liquid cell environment, it is used in a qualitative manner to detect magnetosome chains in hydrated bacteria and to follow magnetite biomineralization at the single-cell level. Iron XRF maps of MTB in liquid were collected with minimal loss of counts compared to dried MTB. Bacteria were sufficiently immobilized and stable in the nano-scanning X-ray beam to measure consecutive XRF maps, allowing for X-ray absorption spectral maps to be collected for individual MTB. When MTB samples were cultured with varied iron concentrations, a change in Fe XRF signal intensity was found to correspond with the difference in magnetite content within individual bacteria. A custom microfluidic device was further constructed to meet the technical requirements of conducting *in situ* experiments “on-chip” in order to follow magnetite biomineralization over several hours.

## Results and discussion

### Nanobeam-scanning X-ray fluorescence imaging and spectroscopy in a liquid cell

**Fig. 1:**
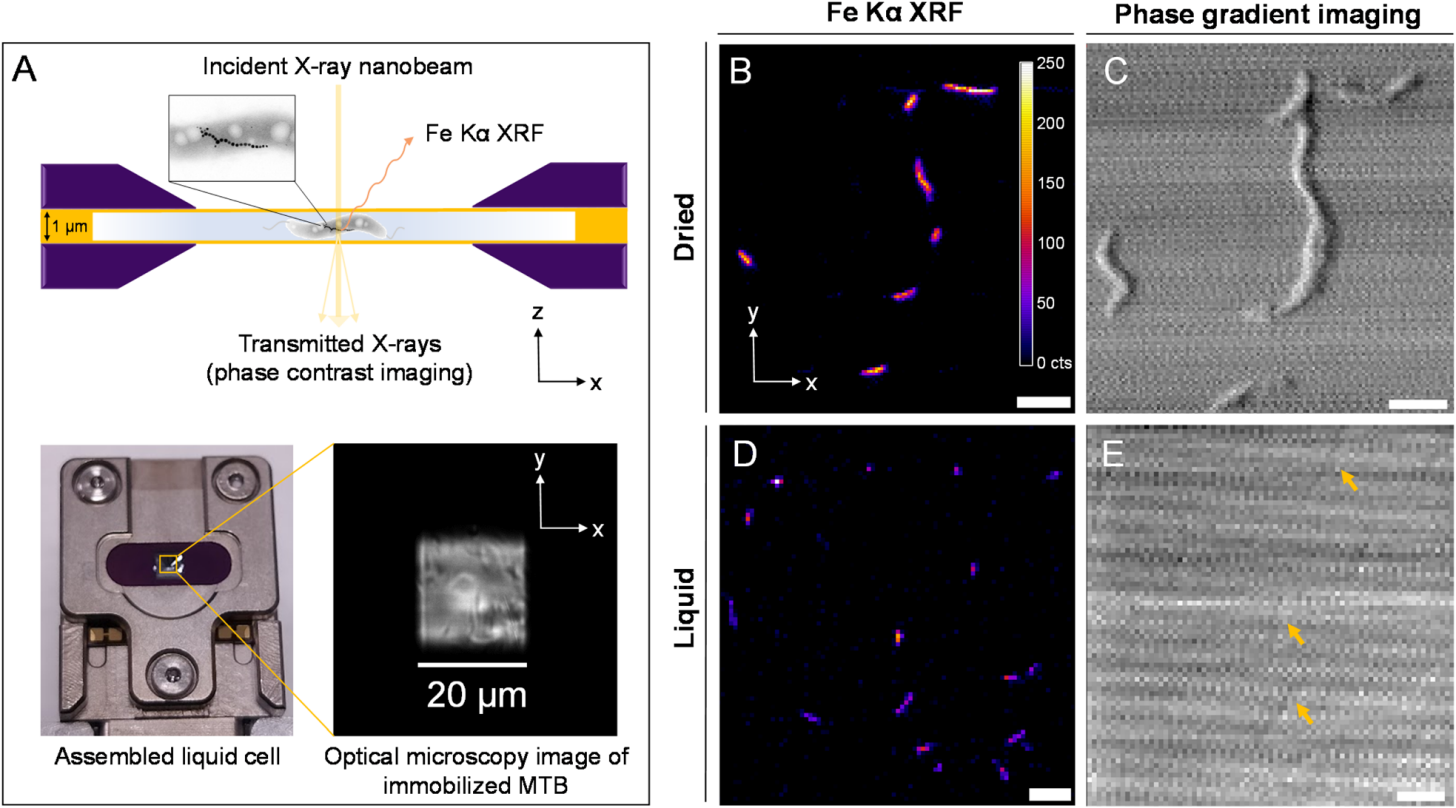
Nanobeam-scanning X-ray fluorescence imaging (nano-XRF) of MSR-1 dried and in a liquid cell. (A) Schematic of a SiN-“sandwich” liquid cell device with immobilized magnetotactic bacteria for nano-XRF measurement (top) and image of the assembled liquid cell with the optical microscopy image of the SiN window region (bottom). MSR-1 measured under dried conditions (B and C) at 100 nm scanning resolution and the liquid cell (D and E) at 200 nm scanning resolution with Fe Kα XRF (B and D) maps and phase gradient (PG) contrast imaging (C and E). The XRF intensity scale (in counts (cts)) in (B) applies to all XRF maps. Arrows in (E) identify weak phase contrast from individual bacteria. Scale bars 2 μm.

Magnetotactic bacteria, strain *Magnetospirillum gryphiswaldense* (MSR-1), were grown under standard conditions (50 μM Fe-citrate) and prepared for loading into a liquid cell containing silicon nitride (SiN) materials (see Materials and methods). A coating of poly-l-lysine (PLL) was applied to SiN materials to immobilize bacterial cells for stable measurement. Fig. 1A depicts the SiN-“sandwich” liquid cell configuration for a nano-XRF measurement and the sample holder used to install the liquid cell in the beam path. X-ray fluorescence was collected in the forward scattering direction as the liquid cell raster scans in the *x*–*y* plane normal to the X-ray nanobeam (Fig. 1A). Scattered X-rays were detected in transmission for phase contrast imaging (see Materials and methods).^23,24^ MSR-1 bacteria measured under dried conditions and in the liquid cell are shown in Fig. 1B–E with Fe Kα XRF and phase gradient (PG) imaging (fitted XRF spectra are presented in Fig. S1†). As demonstrated in our previous work,^20,21^ the majority of the Fe XRF signal originates from magnetosomes. Chain structures are evident in Fig. 1B and D. Lower scanning resolution is used to map a large region of the liquid cell to avoid disturbing immobilized bacteria (higher resolution maps of smaller regions are presented in Fig. S2†). Magnetosome chain structures and appreciable Fe XRF intensities are detectable under both dried and liquid cell conditions. At high X-ray energy, bacterial cell membranes have a weak phase contrast against the media background. Only the faint contrast of some bacterial cells is discernible in Fig. 1E (indicated by arrows).

**Fig. 2:**
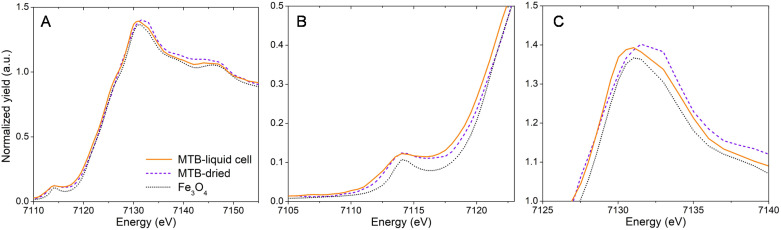
(A) Fe K-edge X-ray absorption near-edge structure (XANES) spectra of MSR-1 bacteria under hydrated conditions (liquid cell) and dried conditions with a magnetite (Fe_3_O_4_) reference. Zoomed in regions for (B) pre-edge (promotion of 1s electrons to unoccupied 3d valence levels) and (C) white-line (promotion of 1s electrons to unoccupied 4p valence levels) features.

Fast mapping times avoided bacteria detachment from the SiN surface. MSR-1 cells were sufficiently stable at intermediate scanning resolution (100–200 nm step size) and exposure times (<60 ms) to allow remapping the same bacterium without it moving position. An energy series of XRF maps could then be collected across the Fe K-edge, producing X-ray absorption near-edge structure (XANES) spectral maps for single cells. Fe K-edge XANES of individual MSR-1 bacteria in the liquid cell are compared with those of dried cells in Fig. 2A (Fig. S3† shows the Fe Kα XRF map of the cell used for XANES spectra, and Fig. S4† shows Fe K-edge XANES spectra from additional individual MSR-1 cells). The general spectral features of MSR-1 dried and in the liquid cell follow the reference magnetite spectrum although some changes are distinguishable. A zoom in of the pre-edge and white-line regions are shown in Fig. 2B and C, respectively. These features reveal information on the average coordination environment and valence state of iron.^25^ The position of the pre-edge feature is similar for all samples though both MSR-1 spectra (dried and liquid cell conditions) have a broader peak than the magnetite reference, with the liquid cell spectrum having a low energy shoulder. The white-line intensity (Fig. 2C) is more dominant at lower energy transitions for the hydrated bacterium, thus shifting the centroid position of this feature to lower energy. This is consistently observed for XANES spectra of other MSR-1 cells in liquid (Fig. S4†). Also notable is the lower absorption energy position (*E*_0_) for the hydrated bacterium compared to the dried conditions and magnetite reference (see Fig. 2B). The collected XANES spectra suggest that more Fe(ii) species are found in the hydrated MTB compared to the dried conditions. This is consistent with previous studies where MTB were found to have ferrous stores in the cell,^26,27^ which are expected to oxidize upon cell dehydration and storage (*i.e.*, dried conditions).^20^

**Fig. 3:**
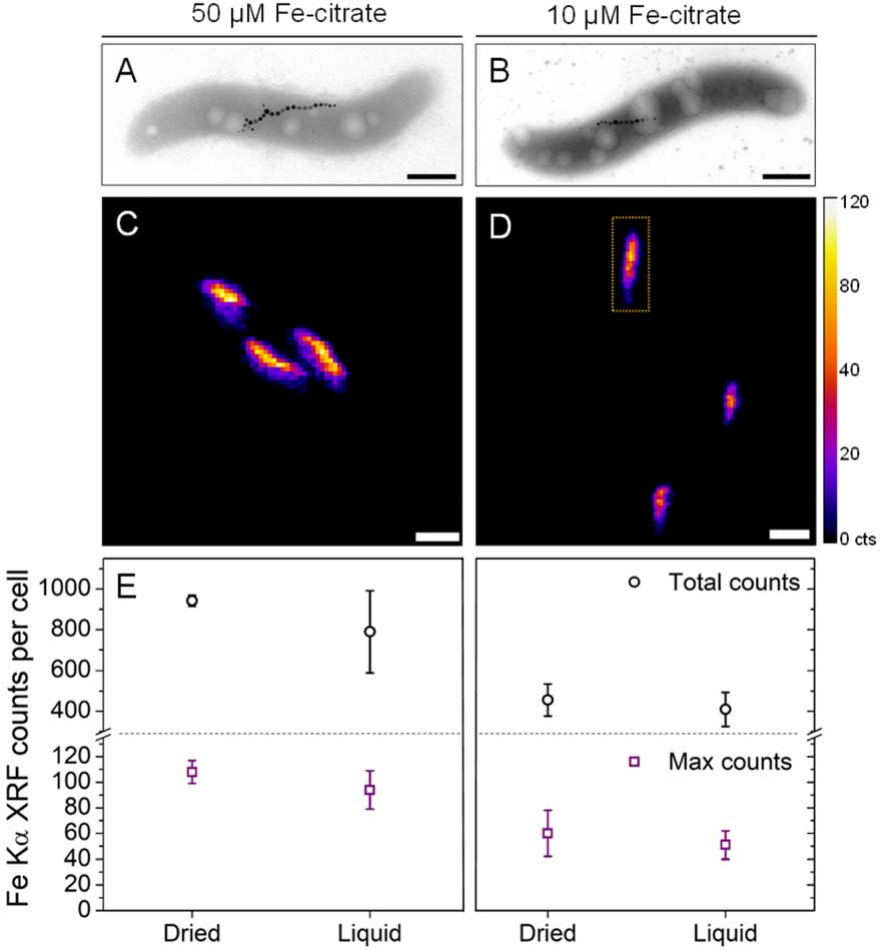
Representative (A and B) transmission electron microscopy images and (C and D) Fe Kα XRF maps of MSR-1 bacteria show a decrease in intracellular magnetite content in response to iron concentration in the culture medium (dried conditions, 50 μM Fe-citrate, left; 10 μM Fe-citrate, right). (E) Extracted total XRF counts per cells and maximum XRF counts per pixel for dried and liquid cell conditions. The XRF intensity scale (in counts (cts)) applies to all XRF maps. The dotted rectangle in (D) shows a typical integration area of 2 μm^2^ for a single cell. Scale bars are 500 nm (A and B) and 1 μm (C and D).

To test the sensitivity of nano-XRF mapping in detecting differences in intracellular iron content, MSR-1 was cultured in 50 μM and 10 μM Fe-citrate to produce different quantities of magnetite. Representative TEM images are shown in Fig. 3A and B (Fig. S5† shows additional images), confirming a reduction in magnetosome size and number when decreasing the iron concentration (Table S1† presents particle size statistics). Fig. 3C and D compare the Fe XRF intensity and distribution for both iron concentrations (additional XRF maps in Fig. S6†). A corresponding decrease in Fe XRF intensity and chain length is found for the 10 μM Fe-citrate condition, as shown in Fig. 3C and D. Two values were extracted from Fe XRF maps: (i) the total counts per bacterium and (ii) maximum pixel counts per bacterium. The former value is related to the total quantity of iron in the region of an individual bacterium (standardized area of 2 μm^2^, see the indicated region in Fig. 3D) and the latter to the size of the largest magnetosome particles or close proximity of two or more magnetosomes, which would result in a higher Fe XRF count for a given pixel. Fig. 3E summarizes these extracted values for both Fe-citrate concentrations under dried and liquid cell conditions (Table S1† shows tabulated values).

TEM analysis (Table S1†) showed that the average number of magnetosomes per cell decreases from 27 to 10 and the average particle diameter decreases from approximately 43 to 35 nm. Using these values and the density of magnetite (5.2 g cm^−3^), and assuming that all particles are spherical, the mass of magnetite per cell was estimated to be ∼4-fold higher for the 50 μM Fe-citrate conditions (Table S1†). However, only a ∼2-fold difference in total Fe XRF counts under both dried and liquid cell conditions is found when comparing concentrations (Fig. 3E and Table S1†). This discrepancy could originate from the fact that nano-XRF measures the total iron content per cell while with TEM only the magnetite content from magnetosomes is detected. Moreover, an additional XRF signal from the integrated area around each cell, from iron species in the cytosolic space, could account for a more similar iron content between samples.^27,28^ For the maximum Fe XRF counts per bacterium, a ∼2-fold difference is found between the two concentrations, which is consistent with the ∼2-fold volume increase in average magnetosome size (calculated from particle diameters in Table S1†).

### Custom microfluidic device

**Fig. 4:**
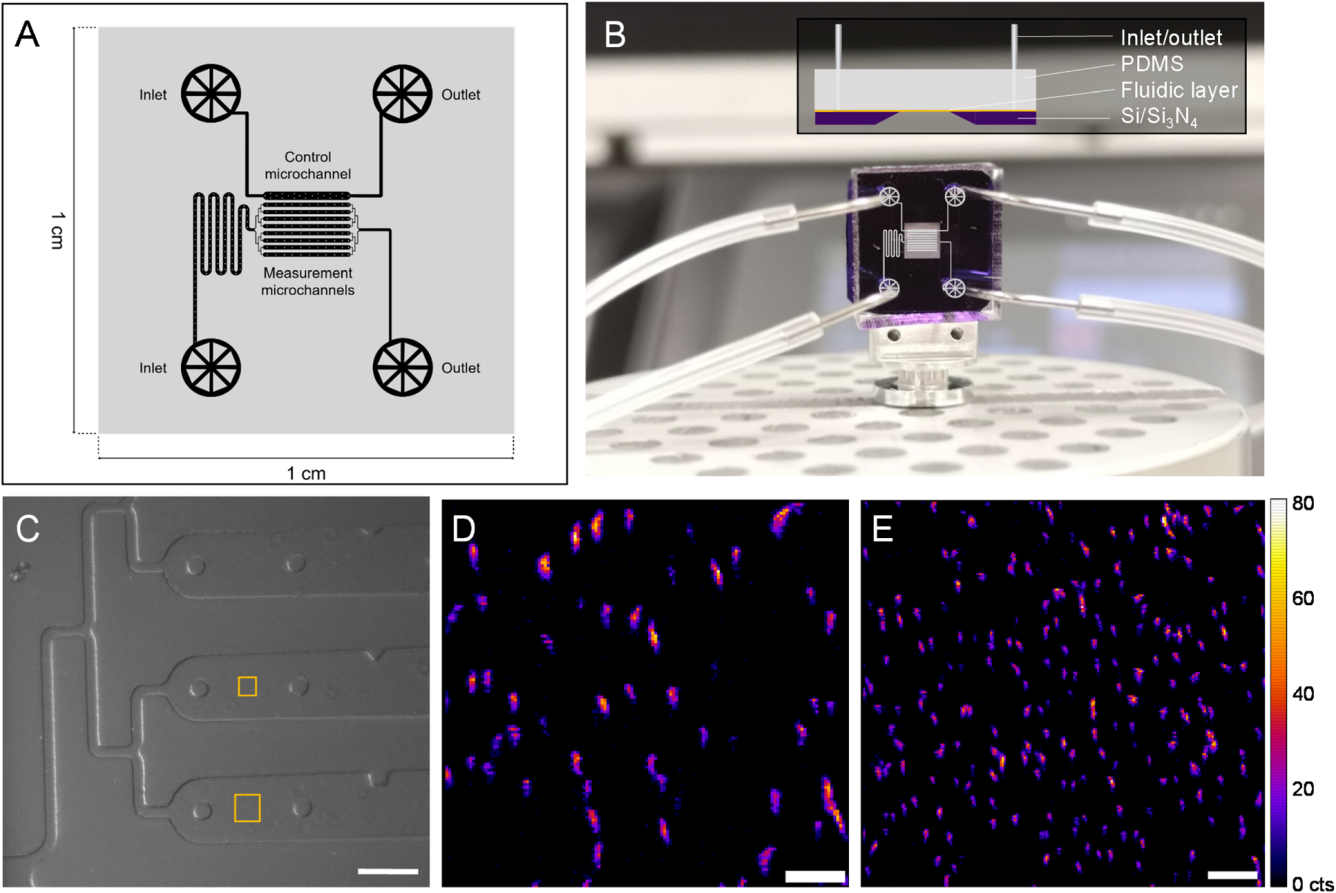
Customized microfluidic device for nanobeam-scanning X-ray fluorescence microscopy (nano-XRF). (A) Layer design for soft lithography preparation of a microfluidic layer in PDMS. (B) Assembled microfluidic device with pins and tubing connected and microchannel design overlaid (inset, side view depiction of the device). (C) In-line optical microscope image of the microfluidic device mounted on a nano-scanning stage at the beamline with squares indicating XRF mapped regions in (D) (smaller square) and (E). Fe Kα XRF mapping of MSR-1 in the customized microfluidic device at (D) 150 nm and (E) 200 nm step size. The XRF intensity scale (in counts (cts)) applies to all XRF maps. Scale bars 100 μm (C), 3 μm (D) and 5 μm (E).

To follow a biological process *in situ* with nano-XRF (*e.g.*, magnetosome formation), the SiN-sandwich liquid cell (Fig. 1) lacks features for experiments that require consecutive mapping over long time periods with living microorganisms. In particular, measurement regions for distinct time points should be isolated in order to minimize the damage inflicted on neighboring cells from scattered X-rays and generated free radicals.^12^ A larger measurable surface and physical barriers between consecutive measurement regions would also facilitate *in situ* experiments. A customized SiN-polydimethylsilane (PDMS) microfluidic device was designed and constructed (see Materials and methods) to meet these demands. Fig. 4A presents the fluidic layer design, which has two independent inlet/outlet systems for running the experiment with 8 channels (100 μm wide and 2000 μm long) and one channel for a control. A master mold was produced to have a height of 10 μm (*i.e.*, 10 μm thick liquid layer) (see Materials and methods). Fig. 4B shows the assembled microfluidic device composed of the soft lithography-produced PDMS material bonded to the SiN membrane substrate, which creates the fluidic layer in between. An overlay of the microchannel schematic in the measurement region is depicted on top of the image in Fig. 4B.

Fig. 4C shows an in-line optical microscope image of the custom device installed in the beamline before nano-XRF mapping. Fig. S7† demonstrates the stability of the custom device mounted on a nano-scanning sample stage as smooth features of wall and support structures are imaged without movement. It also shows the limited Fe XRF background signal from the PDMS material. Similar to the “sandwich” device, the SiN membrane is coated with PLL to immobilize MTB onto the SiN surface. Fig. 4D presents Fe Kα XRF mapping of MSR-1 cells (smaller square in Fig. 4C). Several magnetosome chains of individual bacteria are evident with many aligning in the vertical direction, caused by a magnet in the sample stage below. The custom device has a total measurable surface area of ∼1 × 10^6^ μm^2^ (8 channels × surface area of each channel (100 × 2000 μm^2^)), which is ∼4000 times larger than the measurement region of the “sandwich” liquid cell (Fig. 1). Large mapping regions can be collected with this device, as shown in Fig. 4E, with an area of 40 × 40 μm^2^ that includes more than 150 bacteria. Owing to the versatility of hard X-ray nanoprobe beamlines, our custom microfluidic device was employed to measure hydrated MTB at three synchrotrons (Fig. S8†), including the ESRF, a diffraction limited source (*i.e.*, a fourth generation synchrotron) (Fig. S9†).

### Bacterial cell viability in the X-ray beam

The advantage of the custom device (Fig. 4) for *in situ* experimentation is the large surface area and separated channels. Nevertheless, the impact of beam damage, direct and indirect, on sequentially mapped regions must be anticipated to preserve viability of bacteria over the course of an on-chip experiment. The effect of beam damage on bacteria viability in the custom device was investigated by X-ray mapping a 10 × 10 μm^2^ region (∼5 min of X-ray beam exposure at 10 keV) and performing a live–dead fluorescence assay directly afterwards (see Materials and methods).

**Fig. 5:**
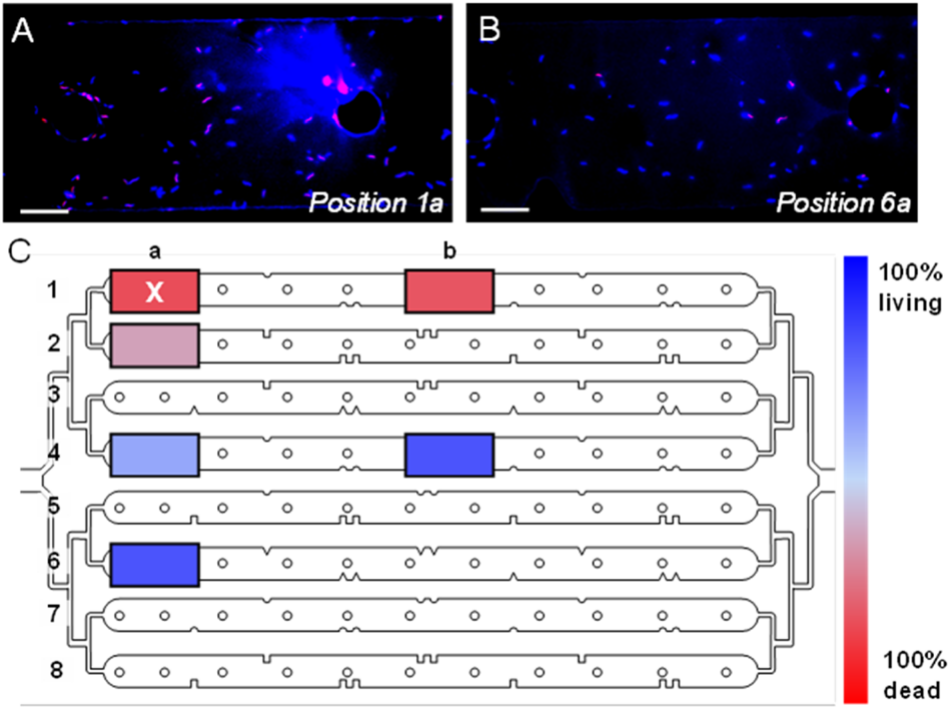
Live–dead fluorescence assay conducted post-X-ray measurement. Epifluorescence microscopy images of (A) X-ray measured channel (top, channel 1 – position a) and (B) distant channel (bottom, channel 6 – position a) (DAPI and PI fluorescence signals are blue and red, respectively). (C) Live–dead assay results corresponding to the position in the microfluidic layer (“X” corresponds to the irradiated region). Scale bars 20 μm.

Fig. 5A and B present optical fluorescence images of the microchannel directly exposed to the scanning X-ray beam and a distant microchannel, respectively, effectively demonstrating the two extremes of membrane-compromised bacteria after X-ray measurement. Membrane-compromised bacteria display red fluorescence from propidium iodide (PI) and are considered “dead”, while DAPI stains all bacteria (Fig. S10† shows images from other microchannels). Fig. 5C summarizes the relative percentage of living and dead bacteria in distinct regions of the fluidic layer (Table S2† shows summarized results). In the X-ray measured region, 85% of cells had compromised bacterial membranes (*i.e.*, 85% dead). It is noted that midway in the same channel (*e.g.*, position 1b) the number of dead cells is comparable. The region directly below the X-ray exposure in the second microchannel (position 2a) had fewer compromised cells. There is considerable cellular damage (>50% dead) in the region likely from X-rays scattered off the PDMS material in the background. This residual dose is reduced over distance as seen from the live–dead results in channels 4 and 6. In these distant microchannels the live–dead result is similar to that when bacteria are first loaded into the microfluidic device (∼85% living, Table S2†). Woehl *et al.* imaged living MTB with TEM using a conventional liquid cell device.^29^ After electron beam irradiation, bacterial cell viability decreased by ∼50%. They attributed a high number of initially inviable cells to the highly restricted liquid cell environment (*i.e.*, compressive forces between SiN membranes). Our custom SiN-PDMS device showed a ∼10% decrease in viable cells compared to the control (Table S2†).

### *In situ* biomineralization experiment

**Fig. 6:**
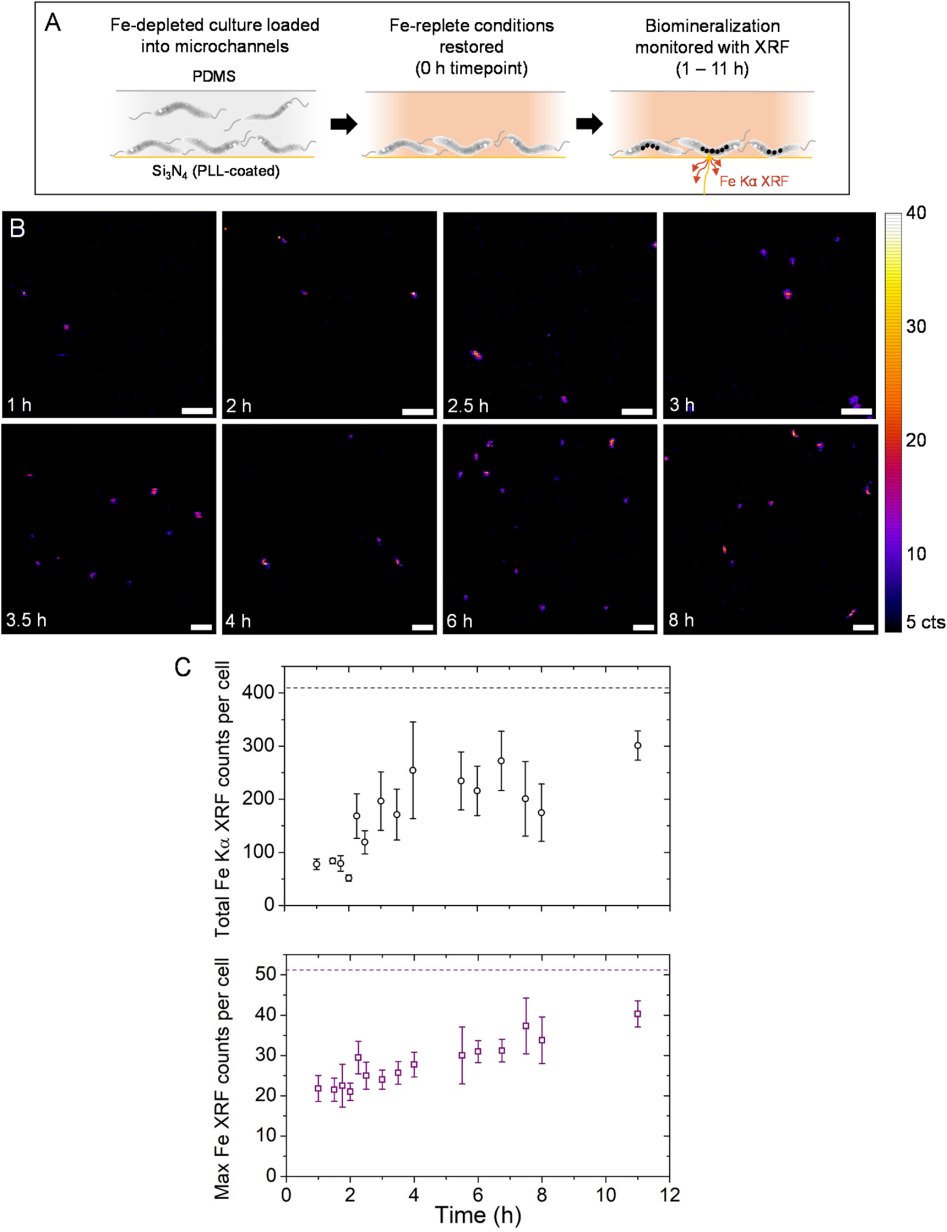
*In situ* magnetite biomineralization experiment conducted in the custom SiN-PDMS microfluidic device. (A) Schematic of the magnetosome induction experiment conducted in the microfluidic device. (B) Fe Kα XRF maps at selected time points from 1 to 8 h over the course of magnetosome formation. (C) Average Fe Kα XRF total counts (top) and maximum count values (bottom) per bacterium for all collected time points. Dashed horizontal lines in (C) refer to the average total Fe Kα XRF counts (top) and maximum counts (bottom) for MSR-1 grown with 10 μM Fe-citrate. The XRF intensity scale applies to all XRF maps. Scale bars 3 μm.

Based on the viability of bacteria after X-ray exposure, measurements during a magnetosome formation experiment were collected diagonally from the top corner to the bottom opposite corner, without measuring the same region twice, in order to minimize X-ray-induced damage to unmeasured bacteria. Also, measurements were conducted starting with bacteria close to the microfluidic outlet, moving to sequential scanning positions upstream in an effort to flush away reactive species generated during X-ray irradiation. Fig. 6A presents a schematic for the induced biomineralization *in situ* experiment (see Materials and methods for full details). Briefly, MSR-1 grown in an Fe-depleted medium for several passages (0 μM Fe-citrate, to suppress magnetosome formation, Fig. S5†) was loaded into the microfluidic device. A short incubation time (no flow from the syringe pump) ensured that MTB would immobilize onto the PLL-coated SiN surface. Afterwards the medium was exchanged for Fe-replete conditions (50 μM Fe-citrate, administered *via* a syringe pump at 1 μL min^−1^), which also washes away non-immobilized MTB. The experiment begins at this point (0 h time point) with a reduced syringe pump flow rate maintained (see Fig. S7† for Fe Kα XRF maps of the microfluidic channel with 50 μM Fe-citrate for the background signal). Fig. 6B shows Fe Kα XRF maps at consecutive time points. Short scanning time per map (∼5 min) and long pauses between consecutive maps (∼15–25 min) were used to limit the total radiation dose. Scanning parameters were identical for all maps, which are the same as those used to extract XRF quantities presented in Fig. 3E.

At 1 h distinct Fe XRF signals were detected, suggesting that magnetosome formation had started (Fig. 6B). Between 2 and 3 h more elongated structures were found, possibly pertaining to magnetosome chain development (*i.e.*, magnetosome particles assembling into linear chains). From 3 h onwards, a higher number of signals were detected per region though chain structures do not appear to develop further. A one-hour pause was made after the 4 h time point and a three-hour pause after the 8 h time point, though the liquid cell device was still receiving fresh media flow. A final time point of 11 h was collected.

The total Fe XRF counts per bacterium and maximum Fe XRF counts per bacterium (both values background subtracted) are plotted in Fig. 6C for all measured time points. The total XRF counts remain low from 1–2 h. After 2 h the average total XRF counts increase and individual bacteria show high variability of XRF intensities (*e.g.*, the 4 h time point). This could be due to a portion of measured bacteria that are mineralizing more slowly or have stopped altogether. On the other hand, the maximum Fe XRF counts per bacterium increase gradually over the course of the *in situ* experiment. This would correspond to either an increase in magnetite nanocrystal size or a higher number of nanocrystals assembled into chain structures. When the liquid cell device was left unmeasured between 8 and 11 h, the 11 h time point showed an increase in both total XRF counts and the maximum XRF intensity. Compared to cultured MSR-1 samples (Fig. 3 and Table S1†), the total XRF counts and maximum counts per bacterium almost reached similar levels to bacteria grown under 10 μM Fe-citrate conditions (see the horizontal dashed lines in Fig. 6C).

At the end of the *in situ* experiment, Fe Kα XRF intensity levels did not match those of complete magnetosome chains in the control samples for 50 μM Fe-citrate conditions (Fig. 3E and Table S1†). This is to be expected as the bacteria were under suboptimal conditions (*e.g.*, immobilized on the substrate, X-ray irradiation, lower temperature) which could slow or limit magnetosome formation. Furthermore, the biomineralization kinetics might be slower due to the lower temperature of the experimental hutch at the synchrotron (typically between 20 and 25 °C) compared to previous time-course biomineralization studies with optimal metabolic temperature (28 °C) and microaerobic conditions (*e.g.*, 1–2% O_2_) for which magnetosomes reach mature stages after 4 to 6 h.^30–33^ By configuring on-stage heating and a gas layer to further reduce oxygen levels, the kinetics on-chip could become comparable to these previous studies though this could introduce other technical issues for device stability on the nano-scanning stage. Nevertheless, the maximum Fe XRF counts per cell increases steadily over the *in situ* experiment, while the total Fe XRF counts increase until 4 h but then vary significantly. This could be interpreted as magnetosome formation activities slowing down after 4 h (*e.g.*, Fe uptake) but chain formation continuing afterwards (magnetosome particles assemble into positions closer to other magnetosomes).

### Limitations and future improvements for measuring biomineralizing bacteria in liquid with X-ray microscopy

Two X-ray scanning techniques were initially considered for measuring MTB in liquid: soft X-ray (∼500–700 eV) scanning transmission X-ray microscopy (STXM) and hard X-ray (∼7000–10 000 eV) scanning X-ray fluorescence microscopy. Both techniques employ nano-sized X-ray beams and 2D raster scan the samples in normal geometry to the incident X-ray beam, but utilize different methods of detection. Although STXM has an advantageous combination of small beam size and lower X-ray energies to provide contrast of biological materials (*i.e.*, C and N K-edges), X-ray absorption contrast of intracellular iron (730 eV) is impeded by the absorption of oxygen (O K-edge at 540 eV), which poses a severe limitation on X-ray transmittance. Fig. S11 and S12† present a “sandwich-like” SiN liquid cell device and STXM mapping of MSR-1 bacteria in liquid. Contrast of bacterial cells and magnetosome chain structures could be achieved at 500 eV and 710 eV, respectively. The contrast of magnetosomes at the latter energy was weak, and further difficulties were encountered when trying to image MSR-1 with few magnetosomes (*e.g.*, 10 μM Fe-citrate conditions) due to the low contrast between the water background and magnetite nanoparticles, despite having a liquid layer thickness of ∼1 μm (Fig. S13†). The technical limitation is in the bright-field approach where smaller magnetite nanoparticles have weak contrast against the cellular background in the “water window” (*e.g.*, <520 eV).

Liquid cell design and measurement strategies to better protect viability should be addressed; otherwise biological processes studied in the laboratory and at the synchrotron will be difficult to correlate. The effect of media flow on guarding cell viability should be pursued. In the tested devices, bacteria began to detach with flow rates >1 μL min^−1^, so higher flow rates could not be used to reduce the indirect X-ray damage experienced in the measured microchannel (*i.e.*, reactive oxygen species). Efficient X-ray dose strategies could also be adopted. This could include a sparse scanning approach,^34^ beam attenuation or employing faster scanning motor stages with shorter detector readout times. The choice of incident ionizing X-ray energy and thus the energy of emitted photoelectrons that scatter through the sample environment during measurement should be considered to minimize damage.^35^ Device design improvements could be the use of many micro-enclosures or microchannels to better isolate individual cells or measurement regions.^18^ Considering the presented custom microfluidic device, X-ray beam damage is expected to be higher due to the XRF emission from the PDMS layer (*e.g.*, Si Kα XRF). A thinner PDMS layer, or more X-ray transparent material such as a cyclic olefin copolymer, could be used to reduce this secondary radiation.

The combination of liquid cell environments and hard X-ray nanoprobe techniques shows promise for many microbiological systems to be investigated in their native-state, and with further developments, amendable to *in situ* experimentation. The nonobligatory vacuum conditions offer the possibility for a wide range of custom liquid cell environments. Nevertheless, with dedicated design and construction, devices have been made for lower energy X-ray microscopy measurements in vacuum environments.^36^ Conducting nano-XRF at a hard X-ray nanoprobe provides access to most biologically relevant metals, which are detectable in a single acquisition (from K to Zn at 10 keV incident energy, as in this work), while X-ray emission from heavier metals can be accessed with incident X-ray energies of 20–30 keV. Core-level XAS spectra can also be collected for 3d metals (K-edge) up to higher 5d metals (L_2,3_-edge) with nanoscale resolution. Beyond absorption and emission spectroscopy X-ray nanoprobe beamlines can employ 2D detectors for X-ray diffraction and scattering techniques,^3^ opening up alternative approaches for studying metal-based nanomaterials in various biological contexts.

## Conclusions

This study demonstrates the capability of nanobeam-scanning X-ray fluorescence microscopy (nano-XRF) to image biomineralizing bacteria in microfluidic environments. Magnetotactic bacteria, which produce magnetite nanocrystals, served as an ideal model for detecting intracellular iron using Fe Kα X-ray fluorescence under liquid conditions. The stability of immobilized bacteria in a liquid cell device allowed for consecutive X-ray fluorescence mapping across the Fe K-edge, enabling the acquisition of Fe K-edge X-ray absorption spectra from individual cells. Radiation-induced damage was assessed using a live–dead assay, revealing localized damage within the device. These findings guided the development of a measurement strategy for conducting a biomineralization experiment “on-chip”. Over an 11-hour period, increasing iron fluorescence signals from individual bacteria suggested the formation of magnetite nanocrystals and the development of chain structures.

The integration of nano-XRF with microfluidic devices provides a powerful method for investigating metal-microbe interactions at single-cell and nanoscale levels under native-state and microfluidic-controlled conditions. Our microfluidic device was constructed to increase the measurable region surface area so that more bacteria could be imaged over several hours of an *in situ* experiment. In principle, our microfluidic layer and device design could be modified to suit another microorganism or *in situ* experiment while remaining compatible with nano-XRF imaging. This approach holds promise for studying metallic particles in biological systems in relevant contexts or in complex media, offering a means to visualize and analyze the dynamics of their interactions. Such capabilities could yield crucial insights into processes such as nanoparticle uptake and intracellular trafficking of metals. As more synchrotron facilities transition to diffraction-limited sources, offering smaller and more intense X-ray beams, optimizing experimental setups and measurement strategies for liquid cell devices becomes increasingly important in order to take advantage of these upgrades. Future innovations in microfluidic device design and radiation dose management will not only enhance native-state imaging of microorganisms but also expand the possibilities for conducting dynamic “on-chip” experiments at synchrotron facilities.

## Materials and methods

### MSR-1 cultivation

*M. gryphiswaldense* (MSR-1) was grown at 28 °C under microaerobic conditions in septum-stoppered glass tubes (1–2% oxygen) in a modified flask standard medium (FSM) for cultured MTB.^37^ Fe(iii)-citrate concentrations of 10 and 50 μM were used to change the quantity of magnetosomes produced. For magnetosome induction experiments, MSR-1 was first grown in a modified FSM medium with very low iron (Fe(iii)–citrate, peptone and yeast extract were omitted from the medium) and under aerobic conditions for three passages.

### Sample preparation for TEM and X-ray microscopy (dried conditions)

Samples of MSR-1 were taken as small aliquots from culture media, centrifuged to remove culture media, and then washed with 0.01 M HEPES and 0.02 M EDTA buffer (pH 7) three times to remove excess metal ions. Finally, cells were washed once with Milli-Q water (18.2 Ω cm). For TEM, 5 μL of washed cells were deposited onto parafilm with a TEM grid placed on top of each drop (film-side down) for at least 15 min. Afterwards, the grids were dried on filter paper. For nano-XRF, 5 μL of washed cells were deposited on a Si_3_N_4_ membrane (Norcada) and left to dry.

### Silicon nitride “sandwich” liquid cell

A liquid cell device (DENS Solutions) commissioned at I14, composed of two silicon nitride (SiN) membranes with 50 nm window thickness and a spacer of 1 μm, was employed to measure MTB in the hydrated state (see Fig. 1A). Poly-l-lysine (0.01% w/v) was used to coat one of the SiN membranes to encourage bacterial cell adhesion (due to electrostatic interactions between the positively charged surface and the negatively charged bacteria) by depositing 1 μL on the SiN membrane and allowing it to dry. 1 μL of MSR-1 cells (at a late exponential phase concentration) was added to one SiN membrane before closing the device with a second SiN membrane on top and securing them within a metal casing that served as the sample holder in the beamline.

### Custom Si_3_N_4_-PDMS microfluidic device

#### Photomask design

The microfluidic channels were designed with AutoCAD 2015 (see Fig. S14† for details). A film photomask of the microfluidic layer design (several copies included in the mask) was purchased from Microlitho to be used for the master mold creation.

#### Master mold creation

A silicon wafer (Siegert Wafer) coated with a patterned photoresist was used to create a master mold for the polydimethylsilane (PDMS) fluidic layer. To prepare the master mold, the silicon wafer was initially baked at 200 °C for 20 min and was allowed to cool down to room temperature. An SU-8 3010 (MicroChem Inc.) thin film was spin-coated onto the wafer to a resulting height of 10 μm, baked and exposed according to the manufacturer recommendations (see Table S3†). The wafer was soaked in mr-Dev 600 (microresist technologies) until full development. The resulting SU-8 film height of the master mold was measured with a white light interferometer (Wyko NT1100). Prior to its use, the master mold was treated with 1*H*,1*H*,2*H*,2*H*-perfluorodecyltriethoxysilane 97% (abcr) to reduce PDMS adhesion. Microfluidic chip fabrication: the microfluidic device was produced *via* soft lithography. Briefly, the PDMS elastomer monomer and a curing agent (Sylgard 184, Dow Corning) were mixed in a 10 : 1 ratio and then degassed to remove air bubbles. PDMS was cast onto the master mold in a square Petri dish to a height of about 2–3 mm. Additional degassing was made to remove air bubbles. The PDMS-covered master mold was then cured at 80 °C for 2 h. It was then allowed to cool down for several hours (overnight), after which the cured PDMS was carefully separated from the master mold and the Petri dish. Individual fluidic layers (1 × 1 cm^2^ slabs) were then separated from each other by cutting PDMS with a blade. For each individual fluidic layer in PDMS, inlets and outlets were punched with a 0.5 mm diameter biopsy punch. Punched PDMS slabs (1 × 1 cm^2^) were plasma-treated in an oxygen plasma cleaner (Harrick Plasma) along with poly-l-lysine-coated Si_3_N_4_ membrane substrates (1 × 1 cm^2^ substrate area with a 3 × 3 mm^2^ window, Silson Ltd). PDMS and Si_3_N_4_ membrane substrates were then bonded to each other by placing them in contact, resulting in the final microfluidic system. This assembly was then heated at 80 °C for 10 min to encourage further bonding.

### Nanobeam-scanning X-ray fluorescence microscopy (nano-XRF)

Measurements were conducted at three X-ray nanoprobe beamlines during the course of this work: I14 of the Diamond Light Source (Oxfordshire, UK), Nanoscopium of the SOLEIL Synchrotron (Paris, France) and ID16B of the European Synchrotron Radiation Facility (Grenoble, France). The majority of data presented herein originates from I14 and Nanoscopium. Samples measured under dried conditions were prepared on TEM grids or Si_3_N_4_ membranes. All measurements were conducted under ambient pressure and temperature conditions using an incident photon energy of 8 or 10 keV for XRF mapping (except for ID16B, 16.5 keV) and a range of 7.0–7.3 keV for collection of Fe K-edge XANES maps (I14 only). PyMCA 5.6.7 software was used to energy calibrate XRF spectra, background subtract, fit XRF sum-spectra and export XRF maps for individual emission lines (*e.g.*, Fe Kα). ImageJ was used to further render XRF maps. Further details for each beamline are given below.

Diamond Light Source, I14: XRF from the sample was collected in front of the sample using a four-element silicon drift detector (RaySpec, UK).^23^ A raster scanning step size of 50–100 nm was used with a dwell time of 30–60 ms to collect high-resolution XRF maps. The focused X-ray beam was ∼60 × 60 nm^2^ in size. A photon-counting Merlin detector (Quantum Detectors, UK) was used in transmission configuration to collect X-ray scattering for differential phase contrast and phase gradient images. Detailed information on how the transmitted signal was transformed, including masking of the beam, background intensity adjustment, and phase integration, was described by Quinn *et al*.^24^ Spectromicroscopy XANES measurements were performed by acquiring 150 XRF maps along the energies of the Fe K-edge. An active drift compensation method was used to maintain alignment between successive scans.^38^ Furthermore, the XANES maps were stacked, aligned, and normalised using the *I*_0_ intensity *via* in-house Python-based scripts. Spectra were extracted from individual cell regions and processed with Athena.^39^

SOLEIL synchrotron, Nanoscopium: the elemental distribution of the sample was measured by XRF under ambient conditions using two silicon drift diode detectors (SDD, VITUS H50, KETEK GmbH) at ∼75° to the incident beam direction.^40^ An incident X-ray energy of 10 keV was used with a beam size of ∼80 nm. Step size and exposure time for higher resolution maps varied between 80 and 150 nm and 50–80 ms, respectively.

European synchrotron radiation facility, ID16B: measurements were conducted under ambient conditions using an incident photon energy of 16.5 keV with 10 Si-drift detectors to measure X-ray fluorescence (XRF).^41^ The focused X-ray beam was measured to be ∼50 nm (FWHM) in diameter. A raster scanning step size of 25 nm was used with a dwell time of 50 ms to collect high-resolution XRF maps.

### Scanning transmission X-ray microscopy

Measurements were conducted at HERMES, a soft X-ray beamline at the SOLEIL Synchrotron (Saclay, France).^42^ A 50 nm Fresnel zone plate was used with measurements under vacuum conditions and at room temperature. An incident X-ray beam energy of 500–730 eV was used to cover both the “water window” and Fe L_3_-edge measurements. A liquid cell holder constructed by Norcada for the HERMES beamline was employed for STXM measurements that use SiN materials with a 400 nm spacer in between to match the thickness of the bacterial cell and minimize the liquid layer. Two SiN membrane materials are used each with 50 nm thickness in the measurement window region. The liquid cell was loaded with a solution of concentrated MSR-1 cells (five times concentrated from late exponential phase culture) with complete magnetosome chains in 0.01 M HEPES medium at pH 7 *via* a syringe pump. Optical microscopy was used to confirm that the measurement window was filled with the medium and bacteria before closing the liquid cell inlet and outlet and loading it into the STXM instrument.

### Transmission electron microscopy

Images were collected using a Tecnai G2 BioTWIN (FEI Company) electron microscope equipped with a charged-coupled device (CCD) camera (Megaview III, Olympus Soft Imaging Solutions GmbH) using an accelerating voltage of 100 kV. Calculation of magnetite quantity per bacterium (Table S1†) was based on the average size and number of magnetosomes per cell measured from TEM images to determine the average volume of magnetite considering the density of bulk magnetite to be 5.2 g cm^−3^.

### Live–dead fluorescence assay

4′,6-Diamidino-2-phenylindole (DAPI) and propidium iodide (PI) were used at a working concentration of 0.5 μg mL^−1^ in HEPES buffer (10 mM) at pH 7. An incubation of at least 15 min was used for effective staining before fluorescence microscopy imaging (for control samples). The bacterial viability after X-ray exposure in the custom liquid cell was performed at the I14 beamline. After X-ray exposure for 5 min at 10 keV (with beam flux similar to that used for nano-XRF mapping), a working concentration solution of DAPI and PI flowed through the device at 0.1 μL min^−1^ for at least 15 min. Afterwards, HEPES buffer without dyes was flowed through the device to wash the cells and device before fluorescence microscopy measurements. A Zeiss AxioImagerM2.m light microscope with epifluorescence was used to collect DAPI and PI fluorescence images. A Zeiss LSM980 laser-scanning confocal microscope was used for assay development and testing.

### Magnetosome formation induction and the *in situ* biomineralization experiment

MSR-1 cultures were grown under iron-limited conditions (Fe-citrate, peptone and yeast extract are removed from FSM medium) with air-exchange and agitation for three passages in order to suppress magnetosome formation (see Fig. S5† for TEM images). Magnetosome formation can then be induced by placing the bacteria under iron-replete conditions (FSM, degassed with nitrogen gas) without air-exchange. To conduct the biomineralization experiment *in situ*, MSR-1 bacteria grown under iron-limited conditions were loaded at a flow rate of 0.1 μL min^−1^ into the custom microfluidic device *via* syringe pump infusion. Tygon tubing (I.D. = 0.5 mm) and metallic pins were used to connect the syringe filled with the growth medium and bacteria to the microfluidic system. The microfluidic device was then incubated at room temperature for at least 15 min to allow bacteria to immobilize on the SiN substrate. The medium was then exchanged for FSM (iron-replete conditions) *via* syringe pump infusion at flow rates of 1 μL min^−1^. The flow rate was then reduced to 0.1 μL min^−1^ for the duration of the experiment. Magnetosome formation was allowed to proceed for several hours. XRF maps were collected starting at the end of the channel and moving upstream (*i.e.*, against fresh media flow to send free radicals and reactive oxygen species to the outlet) and diagonally across the microchannel region.

## Author contributions

Conceptualization: DMC and DF; formal analysis: DMC and LG; funding acquisition: DMC and DF; investigation: DMC, ECD, LG, SS and PEDSR; methodology: DMC, ECD, MAGG and AF; supervision of ECD: TR and DF; visualization: DMC; writing – original draft: DMC; writing – review & editing: all authors.

## Conflicts of interest

There are no conflicts to declare.

## Supplementary Material

SC-016-D4SC08375J-s001

## Data Availability

The data supporting this article are within the manuscript and included as part of the ESI.† Datasets for this article, including optical, electron and X-ray microscopy imaging, are available at Zenedo [https://doi.org/10.5281/zenodo.15095085]. The microfluidic layer design will be made available upon request.
