# Supplementary material for: Imaging biomineralizing bacteria in their native-state with X-ray fluorescence microscopy
Source: Chem Sci. 2025 Mar 3;16(26):12068–79. doi: 10.1039/d4sc08375j (PMC12135071; doi:10.1039/d4sc08375j)
Supplement: SC-016-D4SC08375J-s001 [file SC-016-D4SC08375J-s001.pdf]

Supplementary Information (SI) for Chemical Science.
This journal is © The Royal Society of Chemistry 2025

Supplementary Information:

Imaging biomineralizing bacteria in the native-state with X-ray fluorescence microscopy

Daniel M. Chevrier,*[a,b] Elisa Cerdá-Doñate,[b,c] Lucía Gandarias,[a,d] Miguel A. Gomez-Gonzalez,[e] Sufal Swaraj,[f] Paul E. D. Soto-Rodriguez,[a,g] Antoine Fraisse,[a] Tom Robinson,[c,h] Damien Faivre[a,b]

a. Université Aix-Marseille, CNRS, CEA, UMR7265 Institut de Biosciences and Biotechnologies d'Aix-Marseille, CEA Cadarache, F-13108 Saint-Paul-lez-Durance, France.

b. Max Planck Institute of Colloids and Interfaces, Department Biomaterials, 14424 Potsdam, Germany

c. Max Planck Institute of Colloids and Interfaces, Department Theory & Biosystems, 14424 Potsdam, Germany

d. Depto. Electricidad y Electrónica, Universidad del País Vasco (UPV/EHU), 48940 Leioa, Spain

e. Diamond Light Source, Harwell Science and Innovation Campus, Didcot, OX11 0DE United Kingdom

f. SOLEIL Synchrotron, L'Orme des Merisiers, Départementale 128, Saint-Aubin, 91190 France

g. Instituto de estudios avanzados IUDEA, Departamento de Física, Universidad de La Laguna C/Astrofísico Francisco Sánchez, s/n.E-38203, Tenerife, Spain

h. Institute for Bioengineering, School of Engineering, University of Edinburgh, EH9 3DW Edinburgh, United Kingdom

* Corresponding author: daniel.chevrier@cea.fr

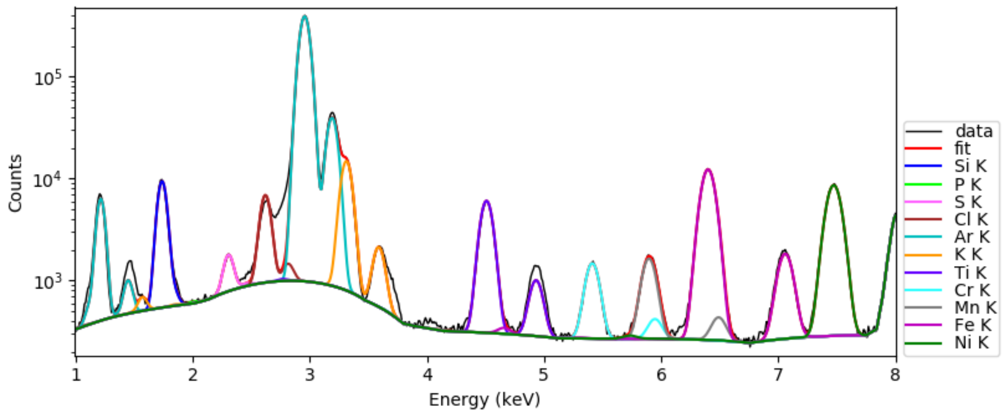

Figure S1. XRF spectra of MSR-1 bacterium in liquid cell with fitted emission peaks.

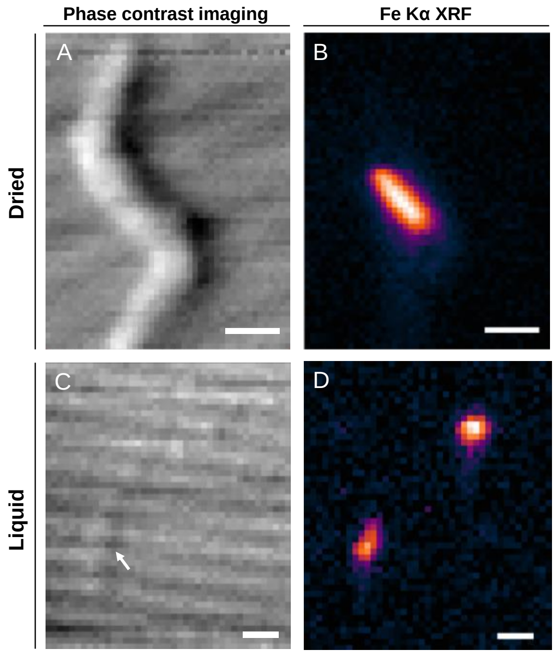

Figure S2. High resolution Fe Kα XRF maps of MSR-1 dried and in liquid (incident X-ray energy 10 keV). Scale bars 1 µm.

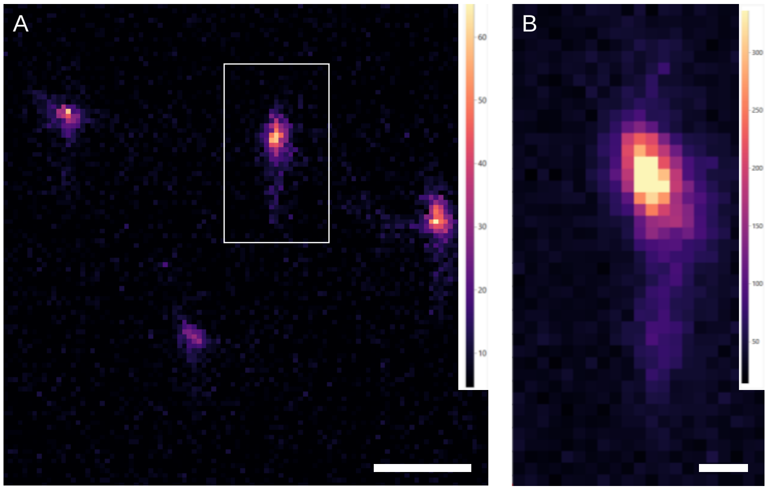

Figure S3. Fe Kα XRF maps of MSR-1 cells in Si_3N_4-“sandwich” liquid cell chosen for Fe K-edge X-ray absorption near-edge spectra (XANES) at low (A) and high (B) scanning resolutions (incident X-ray energy 8 keV). Scale bars: 1 µm (A) and 200 nm (B)

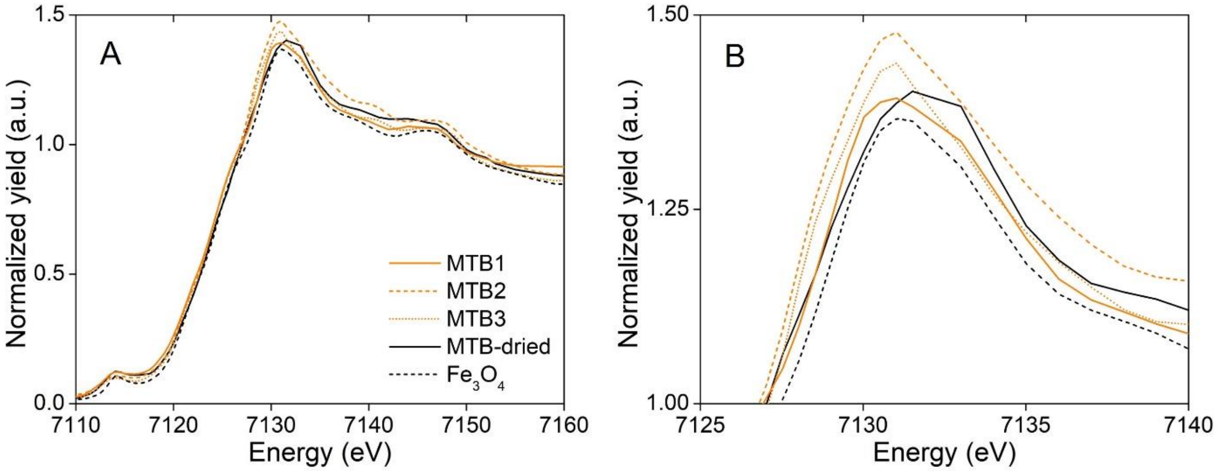

Figure S4. (A) Fe K-edge X-ray absorption near-edge structure (XANES) spectra of MSR-1 bacteria in hydrated condition (liquid cell, MTB-1,2,3), in dried condition and with magnetite (Fe_3O_4) reference. (B) Zoomed in region for white-line feature (promotion of 1s electrons to unoccupied 4p valence levels).

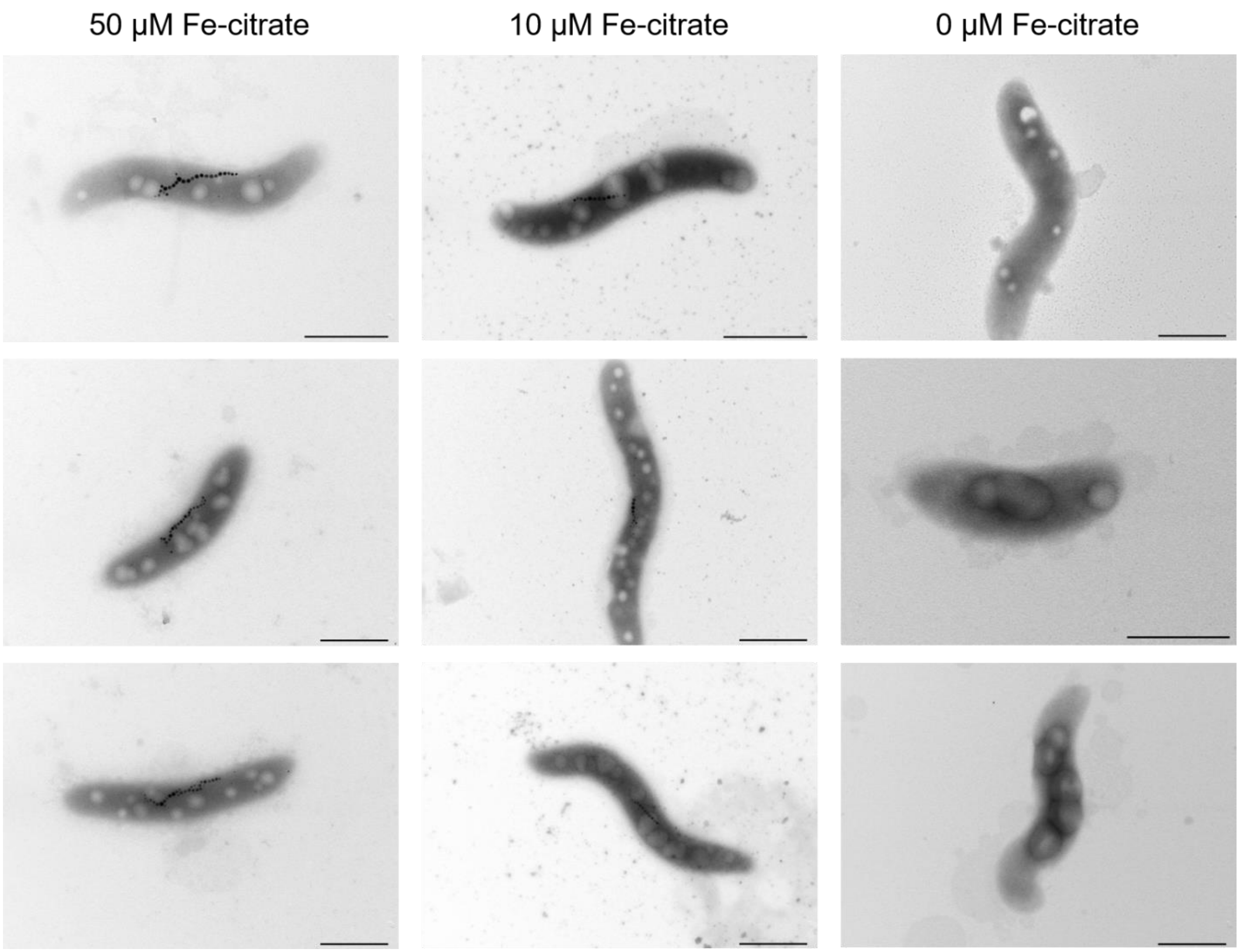

Figure S5. Transmission electron microscopy images of MSR-1 grown under varied concentrations of Fe-citrate. Scale bar 1 μm.

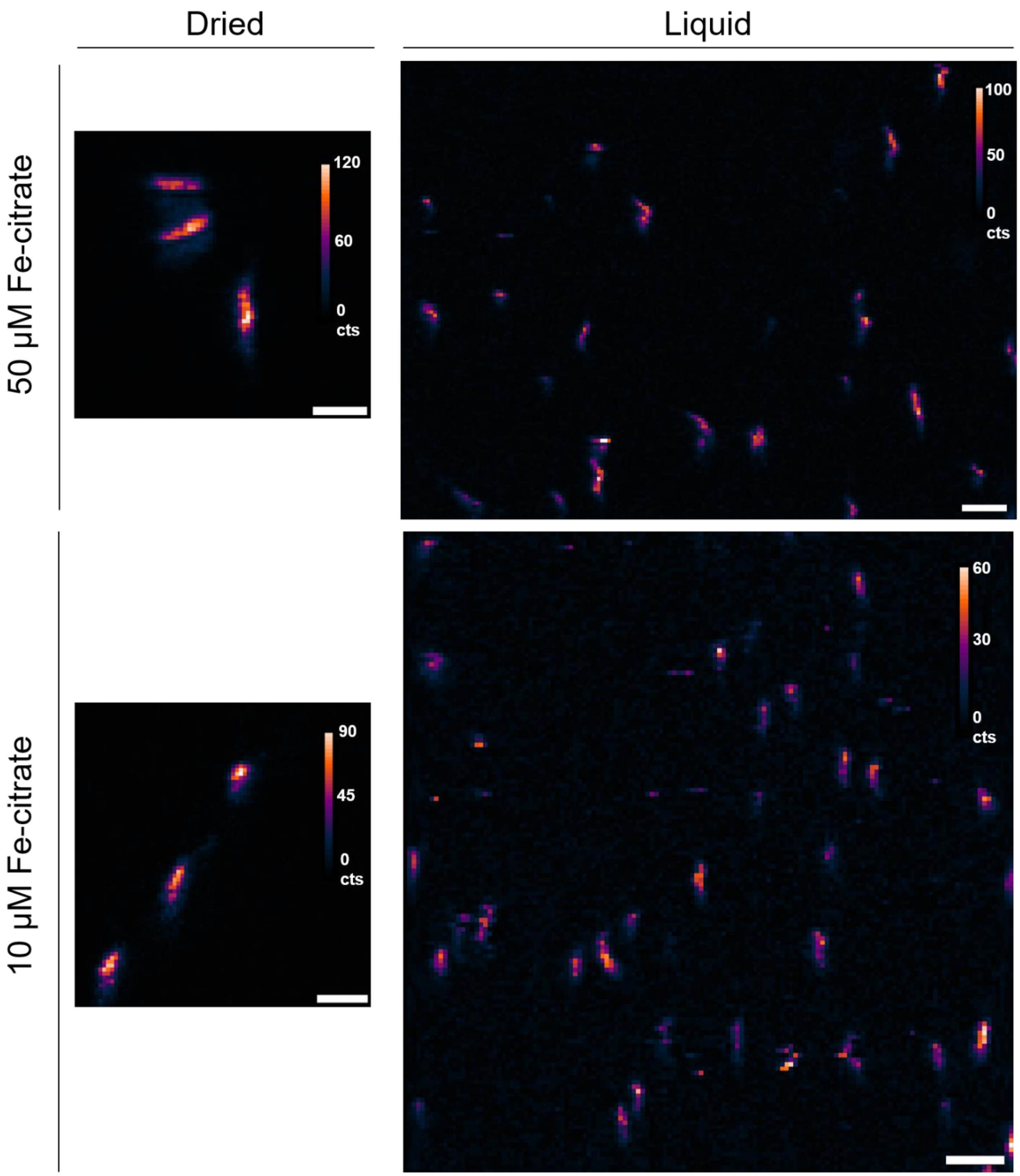

Figure S6. Fe Kα XRF maps of MSR-1 bacteria grown under varied concentration of Fe-citrate dried on substrate and hydrated in liquid cell. Intensity values are in Fe Kα counts. Scale bars are 1 µm for dried condition and 2 µm for liquid cell.

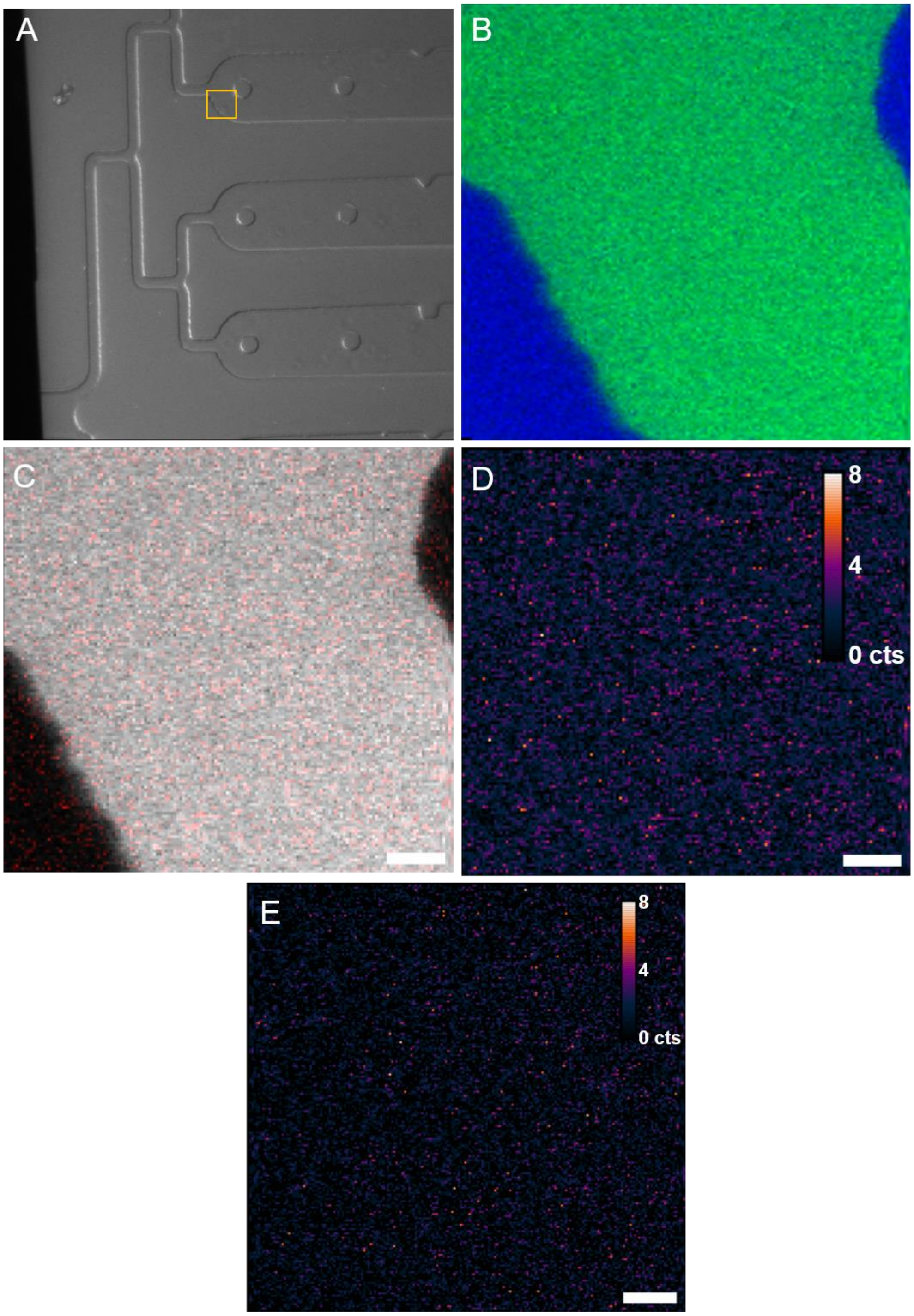

Figure S7. (A) XRF mapped region of device filled with salt water solution. (B) XRF composite map of indicated region in (A) where Si Kα XRF is blue and Cl Kα XRF is green. (C) XRF composite map of region in (A) where Cl Kα XRF is grey and Fe Kα XRF is red. (D) Fe Kα XRF map of indicated region in (A). (E) Fe Kα XRF map of microfluidic device channel filled with 50 µM Fe-citrate medium using same scanning parameters as for *in situ* experiment. Scale bars (C,D) 5 µm and (E) 2 µm.

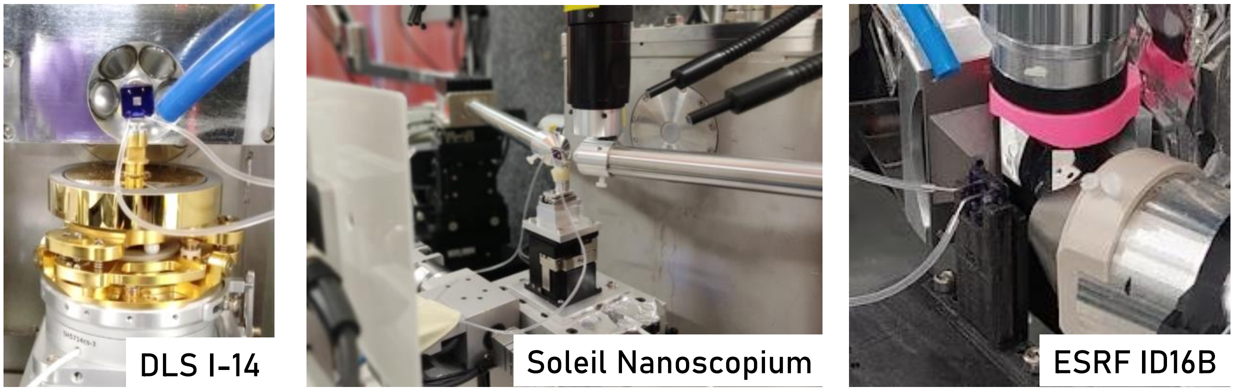

Figure S8. Custom microfluidic device for liquid cell measurements installed at Diamond Light Source I14, Soleil Synchrotron Nanoscopium and ESRF ID16B.

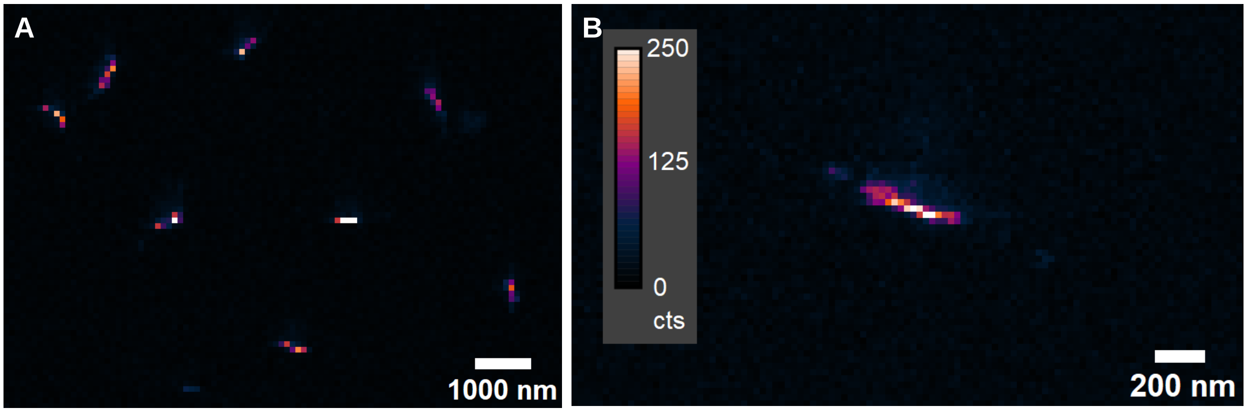

Figure S9. Fe Kα XRF map of MSR-1 (hydrated cell in custom microfluidic device) at fourth generation X-ray nanoprobe at (A) 100 nm scanning resolution and (B) 25 nm scanning resolution.

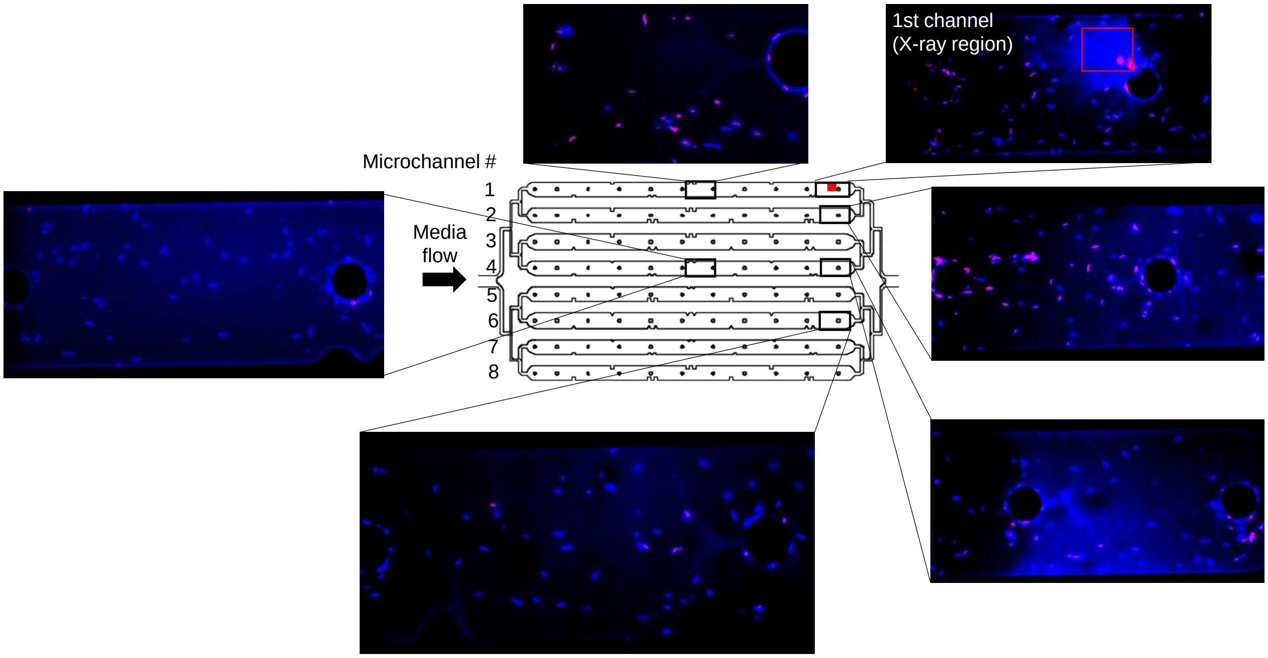

Figure S10. Optical fluorescence images of MTB stained with DAPI and PI for live-dead fluorescence assay after X-ray beam exposure.

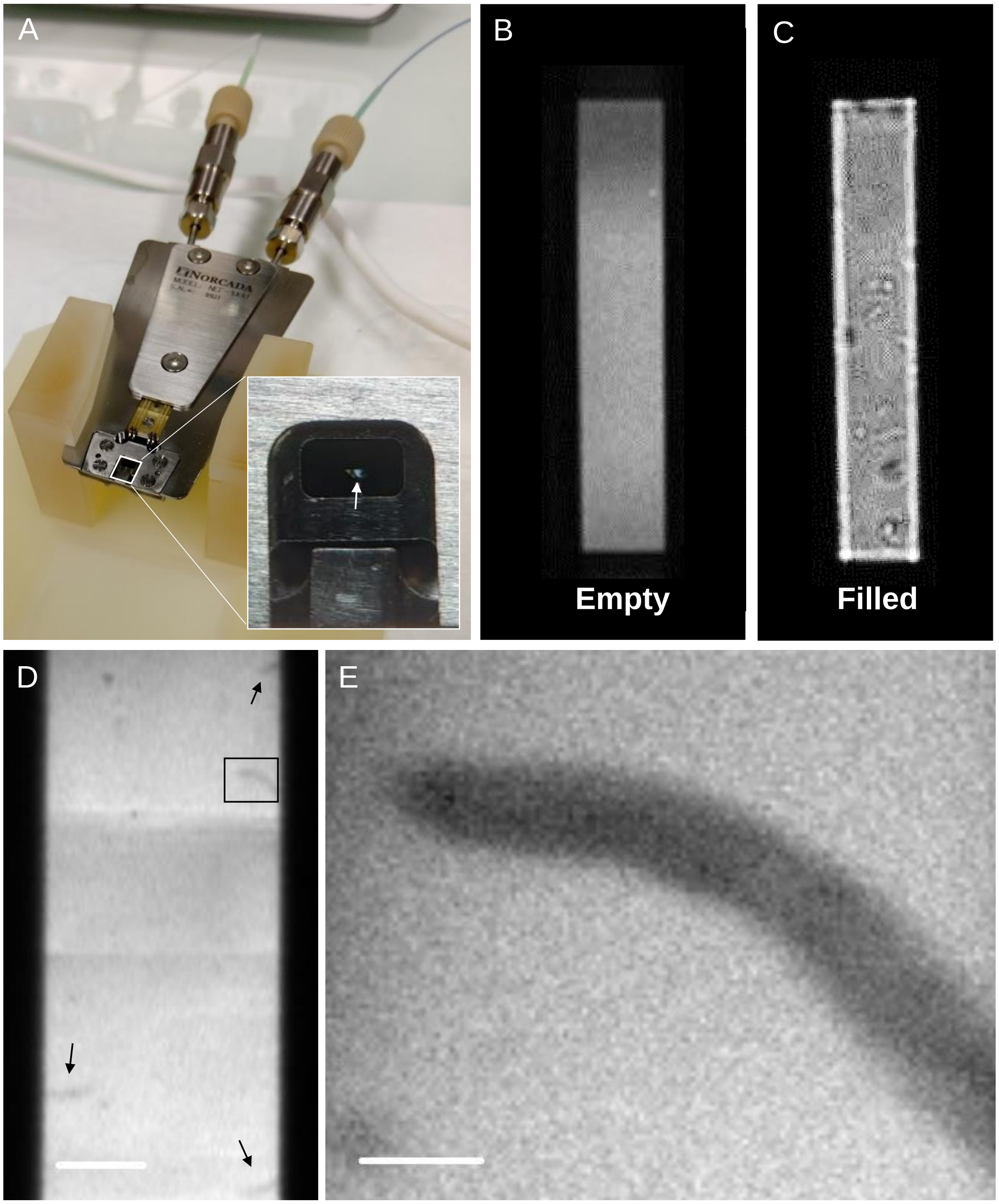

Figure S11. Microfluidic device for soft X-ray scanning transmission X-ray microscopy (STXM) measurements. (A) Microfluidic device fabricated by Norcada with inlet and outlet tubing attached (inset shows stereomicroscope image of assembled and loaded microfluidic device). Light microscope images of Si_3N_4 membrane window empty (B) and filled (C) with MSR-1 bacteria in 0.01 M HEPES medium at pH 7. STXM map of Si_3N_4 membrane window at 500 eV (D) with arrows indicating individual bacteria and box indicating bacterium measured at high resolution (F). Scale bars 5 µm (D) and 500 nm (E).

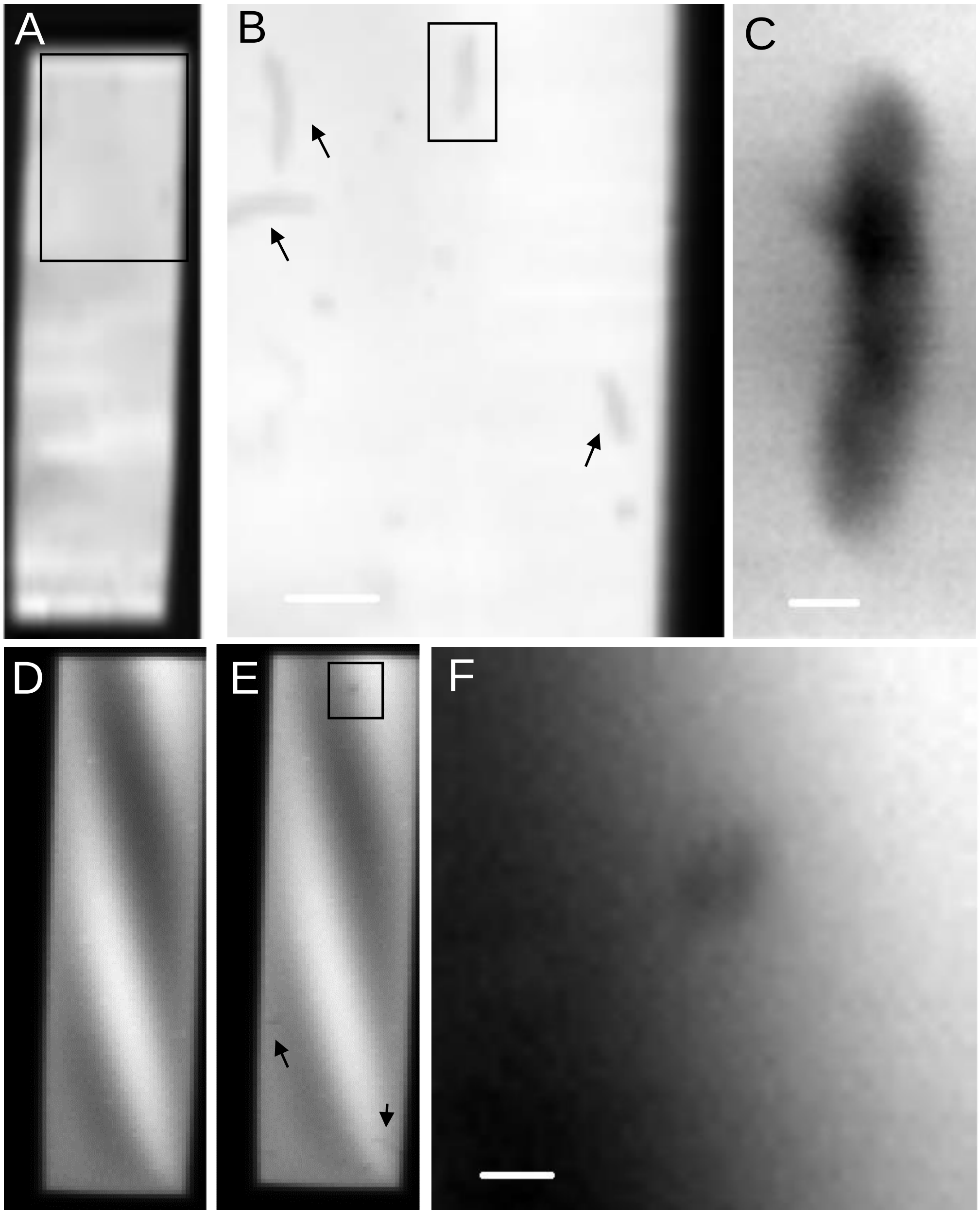

Figure S12. (A) STXM map of Si_3N_4 membrane window region after second loading of MSR-1 in 0.01 M HEPES medium pH 7 with thinner liquid layer (500 eV). (B) STXM map of box region in A with indicators of single bacteria (arrows). (C) High resolution map of single bacterium of box region in B at 500 eV. STXM maps of Si_3N_4 membrane window region at 705 eV (D) and 710 eV (E) with arrows and box indicating single bacterium. (F) High resolution map of box region in (E) of single bacterium at 710 eV with inset Fe L_3 XAS spectrum from energy stack. Scale bars 2 µm (B), 200 nm (C), 500 nm (F).

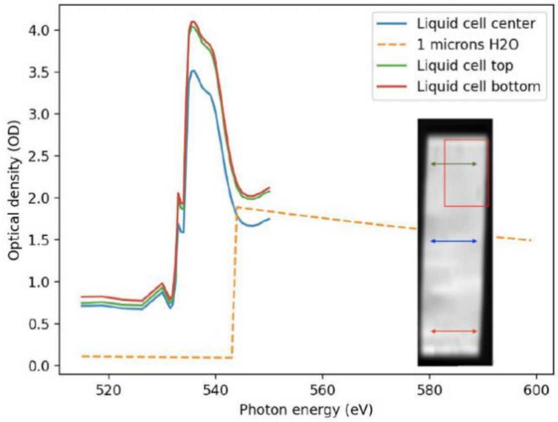

Figure S13. O K-edge XAS spectra integrated over Si_3N_4 membrane window regions indicated by arrows (line scans across regions with no MSR-1). Considering the measured optical density and 0.01 M HEPES media, the liquid layer thickness was calculated to be ~1.1 µm.

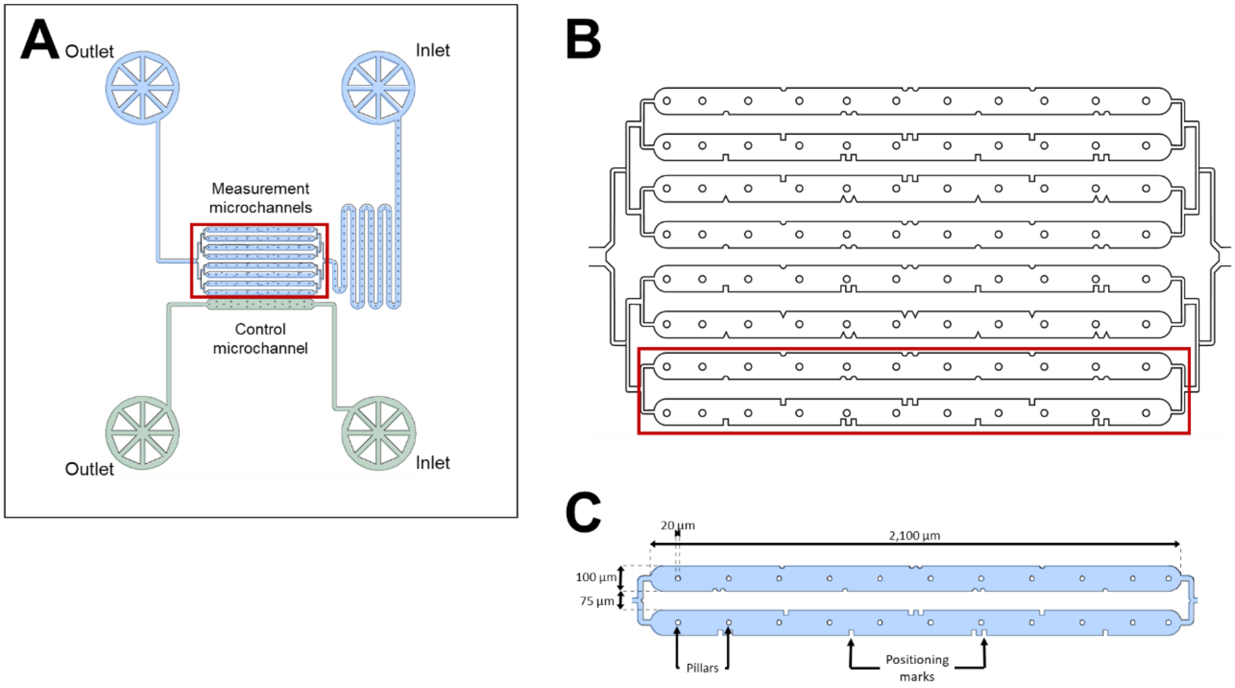

Figure S14. Sketch of the microfluidic channels design. (A) Overview of the whole microfluidic design, which is composed by two independent microchannel systems: the measurement microchannels (blue) and the control microchannel (green). During the course of the presented experiments, only the measurement microchannels (blue) were used. Yet, a control microchannel was created for future measurements as a means of having a control (e.g., magnetite nanoparticles, immobilized bacteria with full grown magnetosome chains) on-chip for time-resolved experiments. The microfluidic system was designed so that it is compatible with Si_3N_4 substrate size (1x1 cm^2). Furthermore, measurement and control microchannels were designed so that they fit on the Si_3N_4 measurement window. (B) Zoom on the measurement microchannel region enclosed by the red box in A. The measurement area has 8 microchannels. (C) Zoom on the microchannel region enclosed by the red box in B. The microchannels were featuring pillars throughout their length to avoid ceiling collapse, as well as positioning marks to facilitate navigation in the system while performing measurements.

Table S1. Magnetosome particle analysis and corresponding X-ray fluorescence signals for MSR-1 grown under culture media with varied concentration of added Fe^{3+}-citrate. Nano-XRF measurements in dried and in liquid cell were conducted using identical incident energy, exposure time and motor step size.

	TEM			nano-XRF	
**[Fe^{3+}] in medium (μM)**	**Average particle diameter (nm)**	**Average number of particles per cell**	**Calculated average mass of magnetite per cell (fg)**	**Total Fe Kα counts per cell**	**Max Fe Kα counts per cell**
**10**	35.1 ± 3.0	10.2 ± 1.8	1.5 ± 0.3	455 ± 79 (dried) 409 ± 84 (liquid cell)	60 ± 18 (dried) 51 ± 11 (liquid cell)
**50**	43.1 ± 2.3	27.2 ± 5.2	6.5 ± 1.4	942 ± 27 (dried) 811 ± 261 (liquid cell)	108 ± 9 (dried) 94 ± 15 (liquid cell)

Table S2. Live-dead fluorescence assay of MTB in custom microfluidic device.

	**Total cells**	**PI-stained cells**	**Non-viable cells (%)**
**Control**	460	29	6.3
**Device incubation 30 min no X-ray exposure**	117	17	14.5
**X-ray exposed channel – position 1a**	102	86	84.3
**X-ray exposed channel – position 1b**	35	6	82.9
**Position 2a**	297	172	57.5
**Position 4a**	96	41	42.7
**Position 4b**	147	33	21.9
**Position 6a**	71	15	21.1

Table S3. Silicon wafer SU-8 3010 spincoating, exposure and post-exposure bake parameters.

#	STEP	PARAMETERS	INSTRUMENT
**1**	**Spincoating**	15 s at 500 rpm,<br>followed by 30 s at 3,000 rpm	WS-650MZ-23NPPB spincoater (Laurell Technologies)
**2**	**Exposure**	5 s to UV	Kloé UV-KUB 3 mask aligner (Kloé)
**3**	**Post-exposure bake**	1 min at 65 °C,<br>followed by 3 min at 95 °C,<br>and 1 min at 65 °C	Heating plates
